# Ultrasonic and Deep Eutectic Solvent for Efficient Extraction of Phenolics from *Eucommia ulmoides* Leaves

**DOI:** 10.3390/foods14060972

**Published:** 2025-03-12

**Authors:** Junliang Chen, Yanhong Song, Xinyu Wei, Xu Duan, Ke Liu, Weiwei Cao, Linlin Li, Guangyue Ren

**Affiliations:** 1 School of Food and Biotechnology, Henan University of Science and Technology, Luoyang 471023, China; chenjl2020@haust.edu.cn (J.C.); 13781255019@163.com (Y.S.); caoweiwei@haust.edu.cn (W.C.); linlinli2020@126.com (L.L.); guangyueyao@163.com (G.R.); 2 College of Agricultural Equipment Engineering, Henan University of Science and Technology, Luoyang 471023, China; wxy15824994023@163.com; 3 School of Food Science and Nutrition Engineering, China Agricultural University, Beijing 100107, China; zhangyanzy112@163.com

**Keywords:** *Eucommia ulmoides* leaves, deep eutectic solvent, response surface methodology, extract

## Abstract

The purpose of this research was to establish an effective method for extracting phenolic compounds from *Eucommia ulmoides* leaves. Seven different deep eutectic solvents (DESs) were prepared, and ultrasonic-assisted technology was employed to optimize the extraction parameters. Factors such as the DES molar ratio, water content, liquid-to-solid ratio, ultrasonic duration, temperature, and power were systematically investigated. The optimal extraction conditions were determined to include a choline-chloride-to-ethylene-glycol molar ratio of 1:4, 30% water content, a liquid-to-solid ratio of 40:1 mL/g, an ultrasonication time of 48 min, a temperature of 53 °C, and ultrasonication power of 60%. Under these optimized conditions, the yields of phenolic compounds and flavonoids reached 17.16 mg/g and 48.23 mg/g, respectively, which were significantly higher (*p* < 0.05) than those obtained by traditional extraction methods. These findings indicate that the use of ultrasonic-assisted DES extraction notably improved the content of active compounds and the antioxidant properties of the extracts. Fourier transform infrared spectroscopy and scanning electron microscopy analyses revealed that this method promotes the release of active compounds by disrupting the integrity of the cell walls. This research offers a theoretical foundation and practical guidance for the efficient utilization and advanced processing of *E. ulmoides* leaves.

## 1. Introduction

*Eucommia ulmoides,* commonly known as Du Zhong, is the only extant species in the family Eucommiaceae. This deciduous tree is highly regarded as a valuable medicinal herb, as documented in the classical Chinese pharmacopoeias Bencao Gangmu and Shennong Ben Cao Jing [1]. Research has demonstrated that the bark of *E. ulmoides* contains bioactive compounds such as iridoids, lignans, and phenolics, which confer a range of health benefits, including antioxidant, anti-aging, anti-inflammatory, antitussive, and hepatoprotective properties [2,3]. However, the bark harvest of *E. ulmoides* requires at least ten years of growth, and inadequate post-harvest management can result in pest infestations and diseases, limiting the sustainable utilization of this resource. Compared to the bark, the leaves of *E. ulmoides* are more abundant and contain comparable bioactive compounds with antioxidant and antimutagenic properties. Leaf extracts are widely utilized for managing hypertension and diabetes and exhibit promising potential in mitigating fatty liver, hypercholesterolemia, oxidative stress, and obesity [4]. Therefore, the effective utilization of *E. ulmoides* leaves not only provides valuable resources for traditional Chinese medicine but also opens up new directions for the development of modern functional foods and natural medicines. Although the bioactive components in *E. ulmoides* leaves hold significant potential, their efficient extraction still faces numerous challenges. Traditional solvent extraction methods are characterized by low efficiency, high energy consumption, and the potential generation of harmful residues, which can lead to environmental pollution. Additionally, high temperatures and prolonged processing times may cause the degradation of bioactive compounds, further limiting their application. Therefore, the development of an efficient and environmentally friendly extraction method has become a key focus of current research.

In recent years, deep eutectic solvents (DESs) have emerged as a novel green solvent, demonstrating significant advantages in the field of natural product extraction. DES is typically composed of a hydrogen bond acceptor (such as choline chloride) and a hydrogen bond donor (such as organic acids, sugars, or alcohols), offering benefits such as simple preparation, low cost, environmental friendliness, and biodegradability [5,6]. Studies have shown that DES has been successfully used to efficiently extract phenolics, saponins, and other bioactive compounds from natural sources such as coffee waste, soapnuts, persimmons, and tea [7,8,9,10]. When combined with ultrasonic-assisted technology, DES extraction methods further enhance extraction efficiency while reducing negative environmental impacts [11].

Although previous studies have explored the application of DES in the extraction of *E. ulmoides* leaves—such as Meng et al. [12], who developed an ultrasonic-assisted DES system for the extraction and separation of chlorogenic acid from *E. ulmoides* leaves, and Luo et al. [13], who investigated the application of DES in the extraction of secondary metabolites from *E. ulmoides* leaves and their seasonal variations—these studies primarily focused on single components or specific extraction conditions. In contrast, this study systematically optimizes the extraction process of phenolic compounds from *E. ulmoides* leaves by integrating ultrasonic-assisted technology with DES and comprehensively evaluates the active component content and antioxidant activity of the extracts. Additionally, this study reveals the mechanism of ultrasonic-assisted DES extraction by analyzing the morphological changes in *E. ulmoides* leaf powder before and after extraction. This research not only provides a theoretical basis for the efficient utilization of *E. ulmoides* leaves but also offers new insights for the innovation and sustainable development of natural product extraction technologies.

## 2. Materials and Methods

### 2.1. Materials and Reagents

*E. ulmoides* leaves were sourced from the Peony Garden at Henan University of Science and Technology. Standards including rutin, gallic acid, chlorogenic acid, quercetin, and kaempferol were obtained from Shanghai Yuan Ye Biotechnology Co., Ltd. (Shanghai, China), while DPPH, ABTS, and 2,4,6-tripyridyl-s-triazine were procured from Shanghai Lan Ji Technology Development Co., Ltd. (Shanghai, China). Folin–Ciocalteu reagent, sodium carbonate, ethanol, acetic acid, sodium acetate, aluminum nitrate, sodium hydroxide, concentrated hydrochloric acid, FeCl_3_·6H_2_O, potassium persulfate (analytical grade), and methanol (chromatographic grade) were supplied by Tianjin Deen Chemical Reagent Co., Ltd. (Tianjin, China).

### 2.2. Experimental Methods

Fresh and disease-free *E. ulmoides* leaves were carefully selected, thoroughly washed, and dried using heat pump drying equipment (GHRH-20, Guangdong Academy of Agricultural Machinery, Guangzhou, Guangdong, China). The drying conditions were set as follows: the temperature of the heat pump dryer was maintained at 50 °C, with an air velocity of 0.5 m/s. The leaves were weighed every 0.5 h until the moisture content of the material was reduced to less than 0.05 g/g on a dry matter basis [14]. The dried leaves were pulverized, sieved through a 100-mesh screen, and stored in a desiccator until further use.

#### 2.2.1. Preparation of Deep Eutectic Solvents

A total of seven distinct deep eutectic solvent (DES) formulations were developed, as detailed in Table 1. The DES formulations were prepared based on established methods from the literature [12,13]. The DES formulations were heated and stirred at 80 °C until a clear, stable, and homogeneous liquid was formed. This mixture was then allowed to cool to room temperature for subsequent use. This preparation method ensures the reproducibility and stability of the DES, which is critical for the efficient extraction of bioactive compounds from *E. ulmoides* leaves.

#### 2.2.2. Extraction Process of Total Phenolics and Flavonoids from *Eucommia ulmoides* Leaves

Precisely weigh 0.1 g of *E. ulmoides* leaf powder and transfer it into a 10 mL centrifuge tube. Add 3 mL of the extraction solvent to the tube, then place it in an ultrasonic bath (KQ3200DE CNC Ultrasonic Cleaner, Kunshan Ultrasonic Instrument Co., Ltd., Kunshan, Jiangsu, China) for extraction. Set the ultrasonication temperature to 50 °C, the power to 60%, and sonicate for 40 min. Subsequently, centrifuge the mixture at 10,000 rpm (11,180× *g*) for 10 min using a centrifuge (TG16-WS High-Speed Centrifuge, Xiangyi Centrifuge Instrument Co., Ltd., Changsha, Hunan, China) and collect 2.0 mL of the supernatant for subsequent testing.

#### 2.2.3. Single-Factor Experiments

Table 2 outlines the single-factor experimental design, which examines the effects of various factors, including the DES molar ratio, water content, liquid-to-material ratio, ultrasonication time, temperature, and power, on the yield of total phenolics and flavonoids extracted from *E. ulmoides* leaves.

#### 2.2.4. Response Surface Optimization Experiment

A response surface optimization experiment was conducted using the Box–Behnken design, which involved four variables and three levels, as outlined in Table 3. The entropy weight method was utilized to assign objective weights to the total phenolic and flavonoid contents, and the corresponding scores were then calculated. Subsequently, response surface analysis was performed on the combined scores.

#### 2.2.5. Determination of Total Phenolic Content in *Eucommia ulmoides* Leaves

The total phenolic content was measured using a modified method based on the approach outlined by Kutlu et al. [15], employing the Folin–Ciocalteu reagent in combination with spectrophotometric detection. A 0.4 mL aliquot of *E. ulmoides* leaf extract, obtained through various extraction methods, was transferred to a 10 mL centrifuge tube. To this, 2.0 mL of tenfold-diluted Folin–Ciocalteu reagent and 3.0 mL of a 10% sodium carbonate solution were added. The mixture was allowed to incubate in the dark for 30 min, after which its absorbance was measured at 765 nm using a spectrophotometer. After measuring the absorbance, the total phenolic content was determined based on the standard curve [14]. The total phenolic content was expressed as milligrams of Gallic Acid Equivalents per gram of dry weight (mg GAE/g DW). The calibration curve equation was y = 0.0008x + 0.0009, with a coefficient of determination (R^2^) of 0.9941. To eliminate the influence of moisture content, the results were converted to a dry matter basis. The moisture content of the samples was determined by drying at 105 °C until a constant weight was achieved. The total phenolic content was then adjusted based on the dry matter content.

#### 2.2.6. Determination of Total Flavonoid Content in *Eucommia ulmoides* Leaves

The total flavonoid content was determined using a modified method based on NaNO_2_-Al(NO_3_)_3_-NaOH, as described by Valcarcel et al. [16]. Rutin was used as the standard flavonoid compound to construct the calibration curve, and the flavonoid content was expressed as milligrams of Rutin Equivalents per gram of dry weight (mg RE/g DW). The calibration curve equation was y = 0.0001x − 0.0305, with a coefficient of determination (R^2^) of 0.9929. A 2 mL aliquot of *E. ulmoides* leaf extract was placed into a 10 mL centrifuge tube, to which 0.2 mL of a 5% NaNO_2_ solution was added. The mixture was then thoroughly mixed. After a 6 min reaction, 0.2 mL of a 10% Al(NO_3_)_3_ solution was introduced and thoroughly mixed, then the reaction was allowed to continue for another 6 min. Finally, 2 mL of a 4% NaOH solution was added. After reacting for 15 min, the volume was adjusted to 5 mL using distilled water, and the absorbance was recorded at a wavelength of 510 nm.

#### 2.2.7. Determination of Main Active Component Content

Accurately weigh 0.4 g of *E. ulmoides* leaf powder into a 10 mL centrifuge tube, add 4 mL of 80% methanol solution, and vortex for 30 s to ensure homogenization. Subsequently, carry out ultrasonic extraction for 30 min, followed by centrifugation at 10,000 r/min for 5 min. The supernatant is subsequently collected, passed through a 0.22 μm membrane filter, and stored at 4 °C for later analysis.

HPLC conditions: The analyses were conducted using a 1260 Infinity HPLC system (Agilent Technologies, Santa Clara, CA, USA) equipped with a ZORBAX SB-C18 column (250 mm × 4.6 mm, 5.0 μm, Agilent Technologies, Santa Clara, CA, USA), maintained at a constant temperature of 30 °C. The mobile phases consisted of a 0.1% phosphoric acid aqueous solution (Phase A) and 100% methanol (Phase D), and a gradient elution was applied.

Gradient Elution Programs: For chlorogenic acid, the elution was performed with a gradient of 30–40% Phase A from 0 to 10 min, and 40–30% Phase A was applied from 10 to 11 min at a flow rate of 1.0 mL/min, with detection conducted at a wavelength of 327 nm. For rutin, the gradient elution employed 40–50% Phase A from 0 to 10 min, followed by 50–40% Phase A from 10 to 11 min, with a flow rate of 0.6 mL/min and detection at 360 nm. For the analysis of quercetin and kaempferol, the elution gradient was initially set at 25–35% Phase A over the first 8 min, followed by a reduction from 35% to 25% Phase A between 8 and 11 min. The flow rate during this process was maintained at 0.8 mL/min, with detection conducted at wavelengths of 371 nm for quercetin and 368 nm for kaempferol, respectively. A 10 μL injection volume was used for all samples.

In the process of compound identification, standard solutions of target compounds with known concentrations were first prepared. These standard solutions, along with the samples, were then injected into an HPLC system, and chromatograms were recorded. The peaks in the sample chromatograms were identified by matching their retention times with those of the standard compounds. The area under each peak was calculated using HPLC software. Quantitative analysis was performed by constructing calibration curves, which were plotted with the concentration of the standard solutions against their corresponding peak areas. The concentration of each compound in the samples was determined by substituting the peak area into the respective calibration curve.

External standards were used for quantification, and calibration curves were constructed for each target compound to ensure accurate measurement. The calibration curves were as follows:

Chlorogenic acid: y = 29,863x + 65.952 (R^2^ = 0.9933)

Rutin: y = 26,727x − 243.61 (R^2^ = 0.9996)

Quercetin: y = 51,057x − 884.49 (R^2^ = 0.9948)

Kaempferol: y = 64,752x − 549.77 (R^2^ = 0.9260)

Here, y represents the peak area, and x represents the content of each active compound. Sample solutions were prepared by dissolving the extracts in the mobile phase and filtering through a 0.22 μm membrane filter prior to injection.

#### 2.2.8. Determination of Antioxidant Capacity

The 2,2-Diphenyl-1-picrylhydrazyl (DPPH) radical scavenging activity was evaluated according to the method described by Liu et al. [17], with some minor modifications to the original procedure. The absorbance of the DPPH stock solution was recorded at 517 nm and adjusted to approximately 1.0. A mixture consisting of 100 μL of *E. ulmoides* leaf extract and 900 μL of DPPH solution was prepared and allowed to react in the dark for 30 min, after which the absorbance was subsequently determined at 517 nm. The DPPH radical scavenging activity of *E. ulmoides* leaves was quantified as the amount of ascorbic acid equivalent per gram of dry weight.

The ferric reducing antioxidant power (FRAP) assay, a widely used method for evaluating total antioxidant capacity, was performed following the method of Rothe et al. [18] with modifications: To prepare the FRAP working solution, 10 mL of 10 mmol/L TPTZ solution was mixed with 10 mL of 20 mmol/L FeCl_3_ solution and 100 mL of acetate buffer. The mixture was shaken well and incubated at 37 °C in a water bath for 30 min. A mixture of 100 μL of *E. ulmoides* leaf extract and 900 μL of FRAP solution was prepared, incubated in the dark for 30 min, and the absorbance was measured at 593 nm. The iron reducing ability of *E. ulmoides* leaves was expressed as the equivalent mass of ascorbic acid per gram of dry weight.

The 2,2′-Azinobis(3-ethylbenzothiazoline-6-sulfonic acid) (ABTS) radical scavenging activity was measured using the method of Li et al. [19] with modifications. The absorbance of the ABTS stock solution was measured at 734 nm and adjusted to approximately 1.0. A mixture of 100 μL of *E. ulmoides* leaf extract and 900 μL of ABTS solution was prepared, incubated in the dark for 30 min, and the absorbance was recorded at a wavelength of 734 nm. The ABTS radical scavenging capacity of *E. ulmoides* leaves was quantified as the amount of ascorbic acid equivalent per gram of dry weight.

#### 2.2.9. Fourier Transform Infrared Spectroscopy Testing

For the analysis, *E. ulmoides* leaf powder, both before and after extraction, was analyzed using the KBr pellet method. The samples were scanned using an IRTracer-100 Fourier Transform Infrared Spectrometer (Shimadzu Corporation, Kyoto, Japan) in transmission mode. The spectra were recorded over a wavelength range from 4000 to 400 cm^−1^, with a resolution of 4 cm^−1^ and 32 scans accumulated for each measurement.

#### 2.2.10. Scanning Electron Microscopy Analysis

The *E. ulmoides* leaf powder, both before and after extraction, was coated with a thin layer of gold using a sputter coater. The samples were then mounted in the chamber of a TM3030Plus Desktop Scanning Electron Microscope (Hitachi High-Technologies Corporation, Tokyo, Japan) to observe the microstructural changes. The analysis was conducted at an acceleration voltage of 5 kV.

#### 2.2.11. Statistical Analysis

Data analysis and processing were carried out using IBM SPSS Statistics (version 25, IBM Corporation, Armonk, NY, USA) and Origin (version 2023, OriginLab Corporation, Northampton, MA, USA). The results from response surface methodology (RSM) were evaluated using Design-Expert software (version 10.0.0, Stat-Ease Inc., Minneapolis, MN, USA). All experiments were performed in triplicate, and the resulting data were averaged before analysis.

## 3. Results and Discussion

### 3.1. Effect of Extraction Solvents on Yield of Total Phenolics and Flavonoids from Eucommia ulmoides Leaves

**Figure 1:**
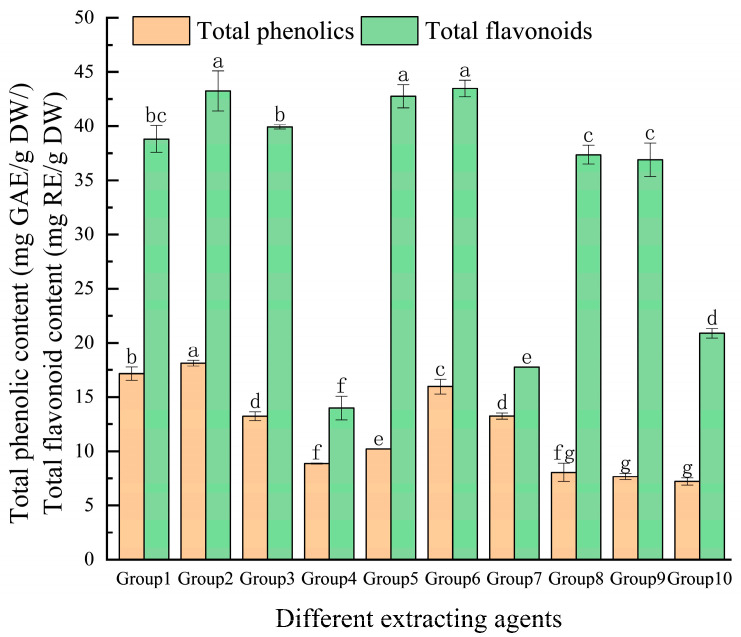
The effect of extraction solvents on the yield of total phenolics and flavonoids from *Eucommia ulmoides* leaves. Total phenolics and flavonoids are expressed as GAE (Gallic Acid Equivalents) and RE (Rutin Equivalents), respectively. The yield is based on DW (dry weight). (Group 1, Group 2, Group 3, Group 4, Group 5, Group 6, Group 7, Group 8, Group 9, and Group 10 represent the different extracting agents DES-1, DES-2, DES-3, DES-4, DES-5, DES-6, DES-7, 80% methanol, 80% ethanol, and water, respectively). Note: Different letters indicate significant differences; the same applies below.

As illustrated in Figure 1, this study assessed the effectiveness of seven deep eutectic solvents (DESs), along with water and two traditional solvents, in extracting total phenolics and flavonoids from *E. ulmoides* leaves. The results revealed that the extraction efficiencies of five deep eutectic solvents (DES-1, DES-2, DES-3, DES-5, and DES-6) were significantly higher than those of the conventional solvents (*p* < 0.05), with DES-2 (choline chloride and ethylene glycol) exhibiting the highest performance. This finding could be attributed to the similarity in polarity between DES-2 and the phenolic and flavonoid compounds present in *E. ulmoides* leaves. According to the principle of “like dissolves like”, solvents with polarity similar to the target compounds more effectively dissolve and release these bioactive components, thereby improving extraction efficiency [20]. It is important to note that although flavonoids are a subclass of total phenolics, the determination of total phenolics and flavonoids in this study was based on Gallic Acid and Rutin Equivalents, respectively. Since these equivalents do not fully represent the entire spectrum of phenolic compounds present in the sample, this could lead to cases where the measured flavonoid content appears higher than the total phenolic content. Additionally, differences in the quantification methods and extraction efficiencies under different treatment conditions may also contribute to this phenomenon. However, this does not affect the overall evaluation of the extraction performance of different solvents. Consequently, DES-2 (a mixture of choline chloride and ethylene glycol) was selected as the optimal solvent for subsequent experiments.

### 3.2. Results of Single-Factor Experiments

#### 3.2.1. Effect of Different Molar Ratios on Extraction Yield of Total Phenolics and Flavonoids from *Eucommia ulmoides* Leaves

**Figure 2:**
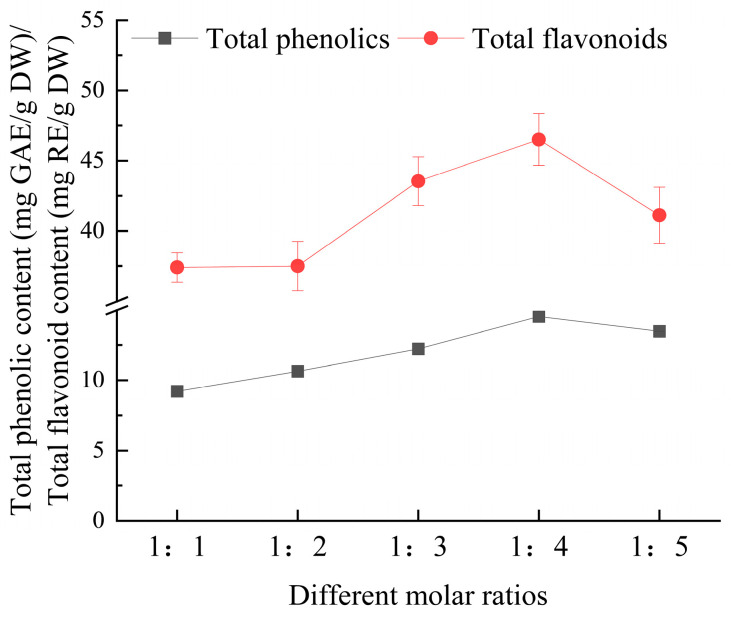
The effect of molar ratios on the extraction yield of total phenolics and flavonoids from *Eucommia ulmoides* leaves. Total phenolics and flavonoids are expressed as GAE (Gallic Acid Equivalents) and RE (Rutin Equivalents), respectively. The yield is based on DW (dry weight).

As depicted in Figure 2, the extraction yields of total phenolics and flavonoids from *E. ulmoides* leaves increased significantly as the molar ratio of choline chloride to ethylene glycol was raised from 1:1 to 1:4, reaching the highest value at the 1:4 ratio. This trend can be attributed to the increased proportion of ethylene glycol, which likely reduced the viscosity of the deep eutectic solvent and optimized its polarity, thereby enhancing the solubility and mass transfer of bioactive compounds [21]. However, when the molar ratio was further increased to 1:5, a decrease in extraction yields was observed. This decline is likely attributed to the excessive amount of ethylene glycol, which may introduce steric hindrance and reduce the solvent–target compound interactions [22]. Therefore, a molar ratio of 1:4 for choline chloride-to-ethylene glycol is recommended for subsequent experiments, as it balances extraction efficiency and solvent properties.

#### 3.2.2. Effect of Water Content on Extraction Yield of Total Phenolics and Flavonoids from *Eucommia ulmoides* Leaves

**Figure 3:**
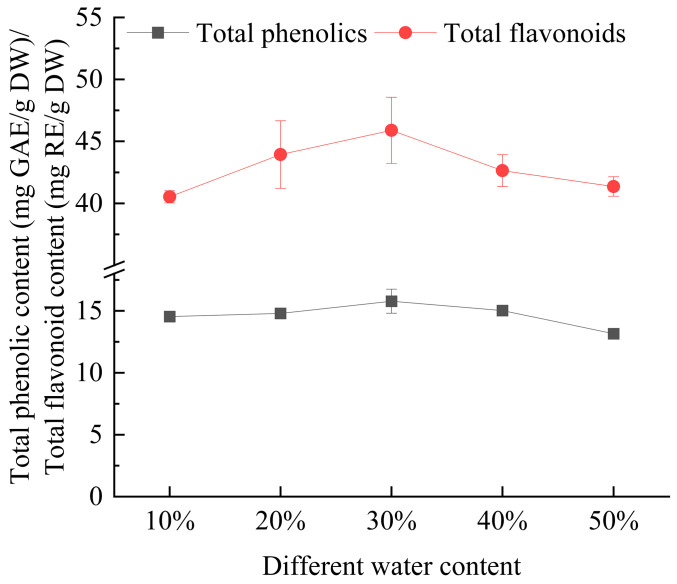
The effect of the water content on the extraction yield of total phenolics and flavonoids from *Eucommia ulmoides* leaves. Total phenolics and flavonoids are expressed as GAE (Gallic Acid Equivalents) and RE (Rutin Equivalents), respectively. The yield is based on DW (dry weight).

As shown in Figure 3, the extraction yields of total phenolics and flavonoids from *E. ulmoides* leaves initially increased and then declined as the water content increased. The highest extraction yields of total phenolics and flavonoids were achieved when the water content was 30%. This could be attributed to the reduction in viscosity and the increase in polarity of the deep eutectic solvent with the higher water content, which facilitated the release of active components and enhanced the extraction yield. When the water content was excessively high, it led to the disruption of intermolecular hydrogen bonds, which weakened the interaction between the solute and solvent [23,24]. This, in turn, resulted in a reduction in the extraction efficiency of total phenolics and flavonoids. A similar trend was observed by Jiao et al. [25] in their study on optimizing the extraction of Perilla leaves using ultrasonic-assisted deep eutectic solvents, where they noted a comparable effect on antioxidant activity.

#### 3.2.3. Effect of Liquid-to-Material Ratio on Extraction Yield of Total Phenolics and Flavonoids from *Eucommia ulmoides* Leaves

**Figure 4:**
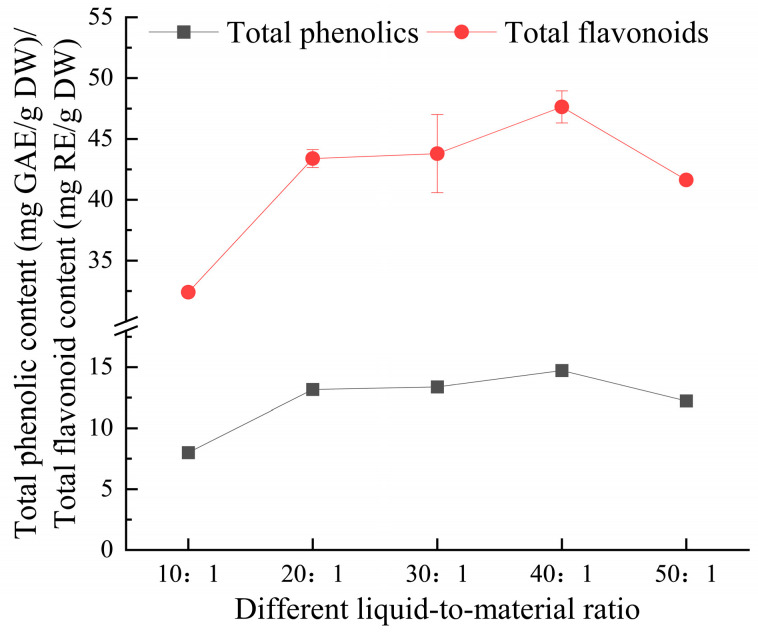
The effect of the liquid-to-material ratio on the extraction yield of total phenolics and flavonoids from *Eucommia ulmoides* leaves. Total phenolics and flavonoids are expressed as GAE (Gallic Acid Equivalents) and RE (Rutin Equivalents), respectively. The yield is based on DW (dry weight).

As illustrated in Figure 4, the extraction yield of total phenolics and flavonoids from *E. ulmoides* leaves initially increased with the liquid-to-material ratio, then decreased after reaching its peak at a ratio of 40:1 mL/g. This is because an increased proportion of extraction solvent facilitated the interaction between the solvent and solute, enhancing solubility and favoring the release of active components. However, once solubility reached a certain level, the diffusion of the solute reached equilibrium, causing some target components to remain undissolved, thus reducing the extraction yield. In their research on the extraction of polysaccharides from Morchella esculenta using ultrasonic-assisted deep eutectic solvents, Pan et al. [26] arrived at a conclusion that is in agreement with the findings of this study.

#### 3.2.4. Effect of Ultrasonication Time on Extraction Yield of Total Phenolics and Flavonoids from *Eucommia ulmoides* Leaves

**Figure 5:**
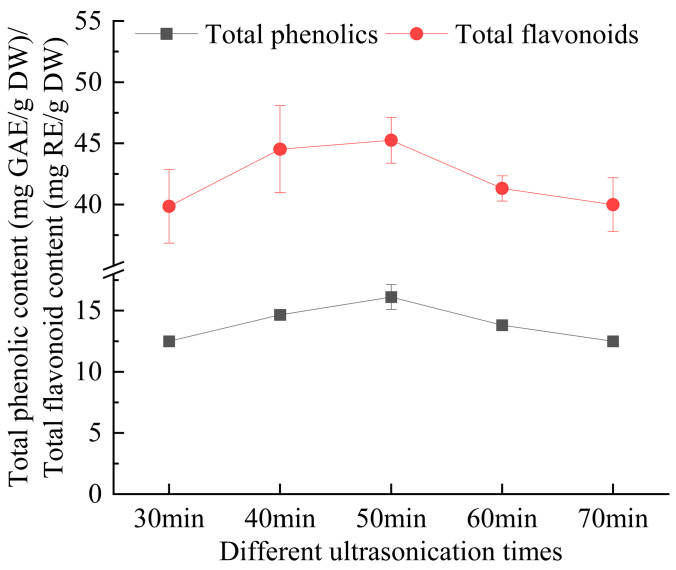
The effect of ultrasonication time on the extraction yield of total phenolics and flavonoids from *Eucommia ulmoides* leaves. Total phenolics and flavonoids are expressed as GAE (Gallic Acid Equivalents) and RE (Rutin Equivalents), respectively. The yield is based on DW (dry weight).

As illustrated in Figure 5, the extraction yield of total phenolics and flavonoids from *E. ulmoides* leaves initially increased with ultrasonication time, reaching a peak at 50 min, before subsequently decreasing as the time continued to rise. The primary reason is that the energy produced by ultrasonic waves increases with ultrasonication time, enhancing the cavitation effect in the solution and thus causing more extensive disruption of the *E. ulmoides* cell walls, which improves the extraction rate. However, continuous ultrasonication can lead to increased temperatures, and the active components might degrade under the dual effects of ultrasonic radiation and thermal effects. Moreover, prolonged extraction can destabilize the structure of compounds such as flavonoids and even cause the degradation of already dissolved phenolics and flavonoids, affecting the extraction efficacy [27,28].

#### 3.2.5. Effect of Ultrasonication Temperature on Extraction Yield of Total Phenolics and Flavonoids from *Eucommia ulmoides* Leaves

**Figure 6:**
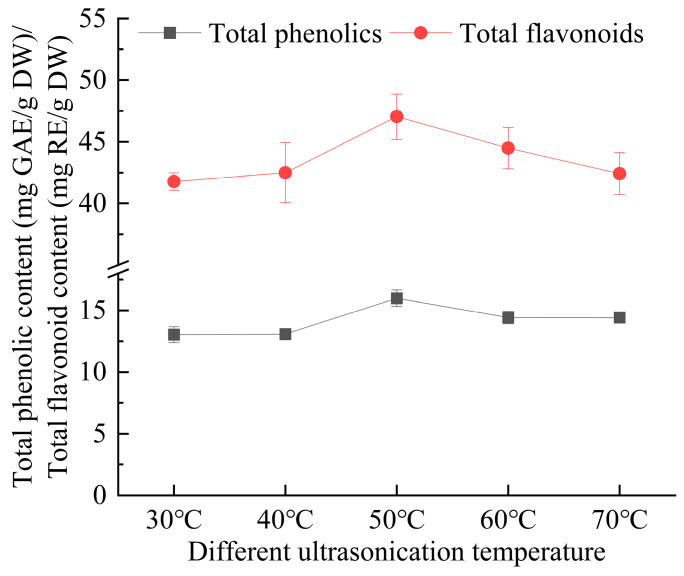
The effect of ultrasonication temperature on the extraction yield of total phenolics and flavonoids from *Eucommia ulmoides* leaves. Total phenolics and flavonoids are expressed as GAE (Gallic Acid Equivalents) and RE (Rutin Equivalents), respectively. The yield is based on DW (dry weight).

As illustrated in Figure 6, the extraction temperature had a significant impact on the efficiency of extracting total phenolics and flavonoids from *E. ulmoides* leaves. The extraction efficiency significantly increased as the temperature was raised from 30 °C to 50 °C, but started to decline once the temperature reached 60 °C. This is because the temperature of the aqueous solution during extraction influences the overall effectiveness of the process. Raising the temperature can enhance the movement of the formed complexes into the extraction solvent, promote better dispersion of the solvent within the aqueous phase, and lower the viscosity of the DES, which facilitates its penetration into the plant matrix, ultimately improving extraction efficiency [29]. Additionally, a higher temperature can enhance the movement of solvent molecules, facilitating greater interaction between the solute and solvent. This, in turn, aids in the diffusion and dissolution of total phenolics and flavonoids. However, further temperature increases may reduce the extraction rate, possibly because the rise in temperature increases the probability of oxidation of the target components and the cavitation effect of ultrasound weakens at high temperatures, thereby affecting the extraction effect [30]. In the study by Baghaei et al. [31], the authors investigated the effect of different temperatures on the extraction and concentration of Co(II) and Ni(II) ions from water samples using DES. They reported a trend that aligns with the findings of this research.

#### 3.2.6. Effect of Ultrasonication Power on Extraction Yield of Total Phenolics and Flavonoids from *Eucommia ulmoides* Leaves

**Figure 7:**
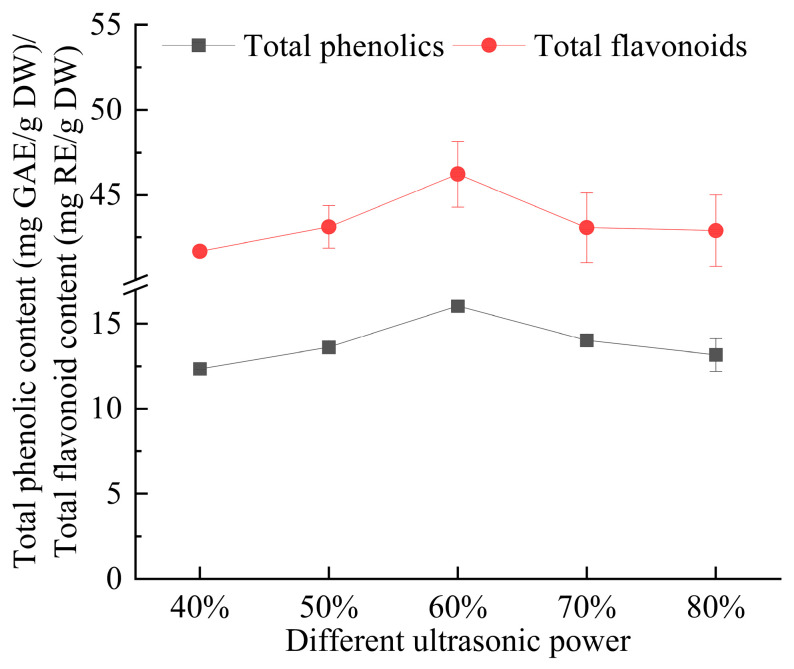
The effect of ultrasonic power on the extraction yield of total phenolics and flavonoids from *Eucommia ulmoides* leaves. Total phenolics and flavonoids are expressed as GAE (Gallic Acid Equivalents) and RE (Rutin Equivalents), respectively. The yield is based on DW (dry weight).

As illustrated in Figure 7, the extraction yields of total phenolics and flavonoids increased steadily as the ultrasonic power increased from 40% to 60%, then slowly declined starting from 70%. This may be because at low ultrasonic power, the effect on cell wall disruption is weak and not conducive to the dissolution of phenolics and flavonoids. With an increase in ultrasonic power, the cavitation effect of ultrasonic vibrations disrupts the plant cell walls and membranes, promoting solvent penetration into the cells [32], facilitating the dissolution of phenolics and flavonoids. However, when the ultrasonic power is increased to a certain level, it may destroy the structure of the active components and the deep eutectic solvent, reducing the extraction yield. Moreover, higher ultrasonic power may cause the dissolution of impurities, obstructing the dissolution of target substances and lowering the extraction yield.

### 3.3. Response Surface Experiment Results

Using the entropy weight method, objective weights were assigned to the total phenolics and flavonoids in *E. ulmoides* leaves, calculating the weight of total phenolics as 0.4635 and flavonoids as 0.5365, with the total score = (total phenolic content × 0.4635) + (total flavonoid content × 0.5365). Taking the total score as the index, the extraction process of *E. ulmoides* leaves was optimized using Design-Expert 10.0.0 software, producing a total of 29 experimental groups, with specific results presented in Table 4.

#### 3.3.1. Regression Model Establishment and Variance Analysis

The experimental data were analyzed through regression fitting and variance analysis, which led to the following model: Y = 33.21 − 0.62A − 1.61B + 2.55C + 2.01D − 1.73AB + 1.75AC + 1.38AD + 2.17BC + 1.04BD − 0.71CD − 6.64A^2^ − 1.72B^2^ − 3.40C^2^ − 7.30D^2^. As indicated in Table 5, the model F-value was 66.83, and *p* < 0.0001, signifying that the model was highly significant, indicating the effectiveness of the model. The lack-of-fit F-value was 4.77, with *p* > 0.05, signifying that the effect was not significant, which implies that the equation models the experiment well. The variance analysis showed that the liquid-to-material ratio, ultrasonication temperature, and ultrasonication power had a highly significant impact on the extraction efficiency of total phenolics and flavonoids from *E. ulmoides* leaves (*p* < 0.01), while ultrasonication time exerted a significant influence (*p* < 0.05). According to the F-values, the four factors affecting the extraction efficiency of total phenolics and flavonoids from *E. ulmoides* leaves are ranked by their influence as follows: ultrasonication temperature > ultrasonication power > ultrasonication time > liquid-to-material ratio.

#### 3.3.2. Response Surface Analysis and Verification Experiment

**Figure 8:**
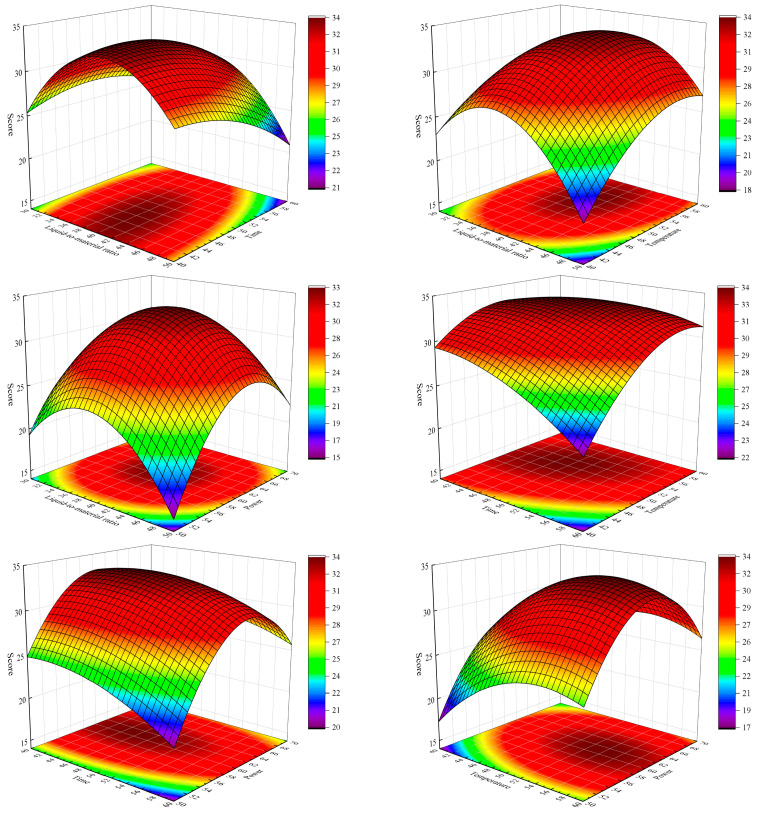
Interaction analysis between factors.

The steepness of the response surface indicates the degree of interaction between the two factors. A steeper surface suggests a more substantial influence of the interaction between these factors on the response value. Figure 8 illustrates the visual representation of the interaction between the factors. The optimal conditions were determined through model fitting using Design-Expert 10.0.0 software, which identified a liquid-to-material ratio of 39.5302:1 mL/g, an extraction time of 48.3407 min, an extraction temperature of 53.2834 °C, and an ultrasonication power of 61.0399%. Under these conditions, the total score reached 33.85. For practical operability, the subsequent verification experiment confirmed the best process conditions as a liquid-to-material ratio of 40:1 mL/g, extraction time of 48 min, extraction temperature of 53 °C, and ultrasonication power of 60%. Three parallel experiments performed under the optimal conditions resulted in a total score of 33.83, which closely matches the predicted value, confirming the accuracy of the model.

### 3.4. Active Component Content

**Figure 9:**
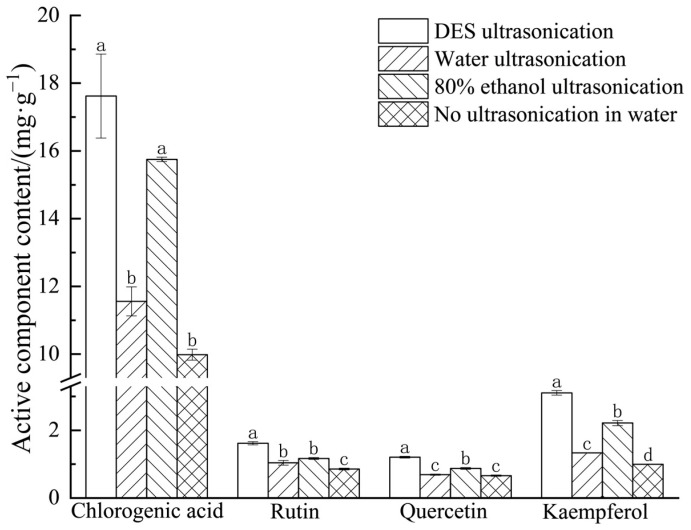
The impact of treatment methods on the content of active components. Note: Different letters in the figure indicate significant differences in the data (*p* < 0.05).

As shown in Figure 9, the various treatment methods notably influenced the levels of bioactive compounds in *E. ulmoides* leaves (*p* < 0.05). The concentration of active components in the extracts from *E. ulmoides* leaves under DES ultrasonication was significantly higher than that under water ultrasonication, 80% ethanol ultrasonication, and non-ultrasonicated water treatment. This might be due to the similarity in polarity between the deep eutectic solvent and the target components, providing better affinity for the extracts. Moreover, the cavitation effect produced by ultrasonication also facilitated the release of active components, thus the DES ultrasonication group had the highest content of active components in the *E. ulmoides* leaf extracts. In their 2020 study on the effect of deep eutectic solvents on the active component content in Plantago seeds, Guo et al. observed that deep eutectic solvents exhibited higher extraction efficiency than traditional solvents.

### 3.5. Antioxidant Capacity Measurement

**Figure 10:**
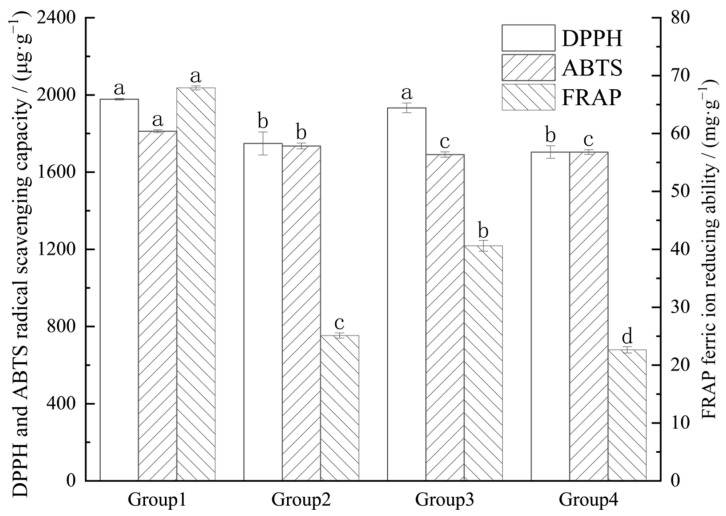
Antioxidant capacity of extracts under different treatment methods. Note: Different letters in the figure indicate significant differences in the data (*p* < 0.05). (Group 1, Group 2, Group 3, and Group 4 represent DES ultrasonication, water ultrasonication, 80% ethanol ultrasonication, and no ultrasonication in water, respectively.)

As depicted in Figure 10, there was no significant difference (*p* > 0.05) in the DPPH radical scavenging activity between the *E. ulmoides* leaf extracts treated with DES ultrasonication and those treated with 80% ethanol ultrasonication. Both of these extracts exhibited significantly higher scavenging activity (*p* < 0.05) compared to those treated with water ultrasonication or without ultrasonication. The FRAP antioxidant capacity and ABTS radical scavenging activity of the *E. ulmoides* leaf extracts treated with DES ultrasonication were significantly higher (*p* < 0.05) compared to those obtained using other treatment methods. Particularly, the FRAP antioxidant capacity, at 67.89 mg/g, was three times that of the group treated without ultrasonication in water. This may be attributed to the ability of DES to improve the dissolution of active compounds, while the cavitation effect generated by ultrasonication aids in breaking down plant cell walls, thereby enhancing the release of bioactive components from *E. ulmoides* leaves. Therefore, the extracts from the DES ultrasonication group exhibited the strongest antioxidant capacity. Xia et al. [32], in their research comparing various traditional solvents, investigated the impact of ultrasonication-assisted deep eutectic solvent on polysaccharide extraction from Anji white tea. They found that, compared to conventional solvents, the Anji white tea treated with this method exhibited enhanced antioxidant activity.

### 3.6. Analysis of Fourier Transform Infrared Spectroscopy Detection Results

**Figure 11:**
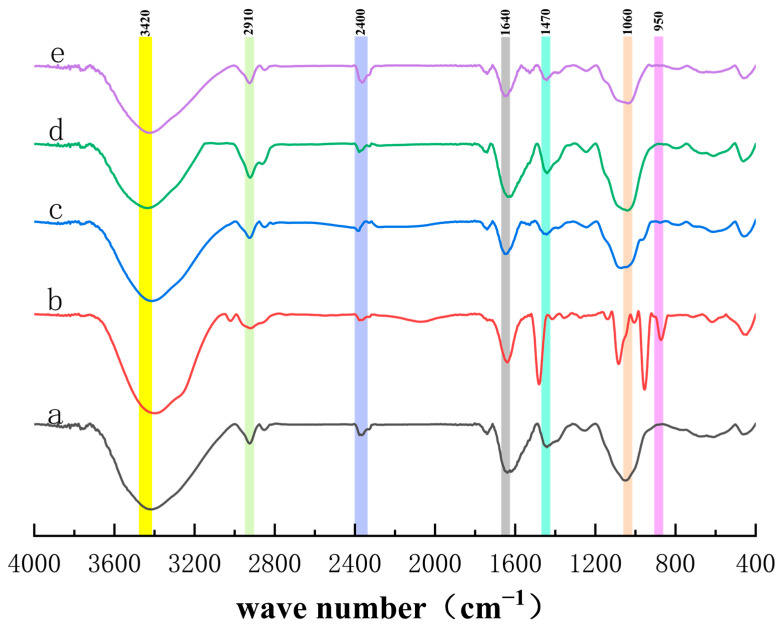
Fourier transform infrared spectra of *Eucommia ulmoides* leaf powder before and after extraction. (a, b, c, d, and e represent untreated *Eucommia ulmoides* leaves, DES ultrasonication, water ultrasonication, 80% ethanol ultrasonication, and no ultrasonication in water, respectively, as different treatment methods.)

Fourier transform infrared spectroscopy was employed to analyze the structural characteristics of *E. ulmoides* leaf powder before and after treatment, focusing on the variations in infrared spectra resulting from different processing methods. As illustrated in Figure 11 (a), the untreated *E. ulmoides* leaf powder displayed a prominent broad absorption band around 3420 cm^−1^, attributed to O-H stretching vibrations. Additionally, a strong C-H stretching vibration was observed near 2910 cm^−1^, along with stretching vibrations of double bonds at approximately 1640 cm^−1^ and pronounced C-H in-plane bending vibrations near 1060 cm^−1^. Comparing Figure 11 (c,e), the intensity of the peak near 3420 cm^−1^ increased while the intensity of the peak near 2400 cm^−1^ decreased in *E. ulmoides* leaf powder after ultrasonication. This suggests that ultrasonic treatment enhances the exposure or release of hydroxyl compounds, likely due to the disruption of hydrogen bonds within the plant matrix. Comparing Figure 11 (a,c,d), the infrared spectra of the *E. ulmoides* leaf powder treated with water and 80% ethanol were similar to that of the original *E. ulmoides* leaf powder, indicating that water and 80% ethanol had no impact on the structure of *E. ulmoides* leaf powder. In comparison to Figure 11 (a,b) revealed double bond stretching vibrations around 1470 cm^−1^ and C-H out-of-plane deformation vibrations near 950 cm^−1^. The appearance of these new peaks suggests significant structural modifications in the *E. ulmoides* leaves, likely resulting from the interaction between the ultrasound-assisted deep eutectic solvent (DES) and the plant matrix, as well as the efficient extraction of bioactive compounds such as phenolics and flavonoids. Specifically, the unique solvent properties of DES combined with the synergistic effects of ultrasonic treatment may have disrupted the plant cell walls, facilitating the release of target compounds while altering intermolecular interactions within the leaves. These structural changes are reflected in the new vibrational features observed in the infrared spectra. Furthermore, these modifications confirm that ultrasound-assisted deep eutectic solvent treatment enhances the extraction efficiency of bioactive compounds by altering the structural composition of *E. ulmoides* leaves. 

### 3.7. Microstructure Analysis

**Figure 12:**
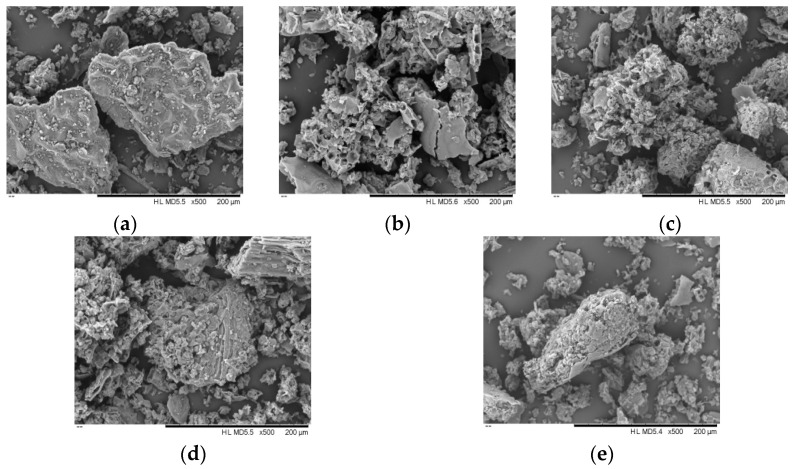
Scanning electron microscopy images of *Eucommia ulmoides* leaf powder before and after extraction. (**a**–**e**) represent untreated *Eucommia ulmoides* leaves, DES ultrasonication, water ultrasonication, 80% ethanol ultrasonication, and no ultrasonication in water, respectively, as different treatment methods.

Morphological changes on the surface of *E. ulmoides* leaf powder were analyzed using scanning electron microscopy, both before and after extraction with ultrasonication-assisted deep eutectic solvent. Figure 12a–e, respectively, show the microstructures of *E. ulmoides* leaf powder before extraction, after extraction with ultrasonication-assisted deep eutectic solvent, ultrasonication-assisted water, ultrasonication-assisted 80% ethanol, and without ultrasonication in water. Figure 12a reveals that the surface of the unextracted *E. ulmoides* leaf powder is poreless and smooth. Figure 12b shows that the surface structure of the leaf powder post-extraction exhibits noticeable pores and cracks, indicating that the deep eutectic solvent can disrupt plant cell walls. Wu et al. [33] utilized scanning electron microscopy to examine the morphology of raw sunflower disc materials and samples extracted with different solvents, aiming to investigate how various extraction methods influence surface structures. Their findings indicated that samples treated with DES exhibited significant surface damage. Figure 12c,d indicate that extraction with water and 80% ethanol also resulted in pores on the surface of *E. ulmoides* leaf powder, but with fewer and less dense pores compared to the microstructure after deep eutectic solvent treatment. Comparing Figure 12c,e, the structure of *E. ulmoides* leaf powder without ultrasonication in water is compact, with only slight surface flaking, suggesting that the cavitation effect of ultrasonication can effectively rupture the cell walls and membranes of *E. ulmoides* leaves, exposing active components and facilitating the extraction of active ingredients.

## 4. Conclusions

Using ultrasonication-assisted deep eutectic solvent (DES) technology, this study effectively extracted phenolic and flavonoid compounds from *E. ulmoides* leaves with high efficiency. Compared with traditional solvents, DES demonstrated significant advantages in enhancing the active component content and antioxidant capacity. Infrared spectroscopy and microstructural analysis revealed that DES effectively altered the microstructure of *E. ulmoides* leaves, promoting the release of active components. This study introduces a novel and sustainable extraction technique, while also paving the way for the wider application of *E. ulmoides* leaves in the medical and food sectors. With its sustainability, efficiency, and environmental benefits, this method shows great potential for application in the field of natural product extraction.

## Figures and Tables

**Table 1:** Various types of deep eutectic solvents.

Group	Components of Deep Eutectic Solvent			
	Hydrogen Bond Acceptor	Hydrogen Bond Donor	Molar Ratio	Water Content
DES-1	choline chloride	lactic acid	1:2	30%
DES-2	choline chloride	ethylene glycol	1:3	30%
DES-3	choline chloride	urea	1:2	30%
DES-4	betaine	lactic acid	1:2	30%
DES-5	betaine	ethylene glycol	1:3	30%
DES-6	choline chloride	glycerol	1:2	30%
DES-7	choline chloride	citric acid	1:2	30%

**Table 2:** Single-factor experiment design.

Number	A: Molar Ratio	B: Water Content/%	C: Liquid-to-Material Ratio/(mL·g^−1^)	D: Ultrasonication Time/min	E: Ultrasonication Temperature/°C	F: Ultrasonication Power/%
1	1:1, 1:2 1:3, 1:4 1:5	30	40:1	50	50	60
2	1:4	10, 20, 30, 40, 50	40:1	50	50	60
3	1:4	30	10:1, 20:1, 30:1, 40:1, 50:1	50	50	60
4	1:4	30	40:1	30, 40, 50, 60, 70	50	60
5	1:4	30	40:1	50	30, 40, 50, 60, 70	60
6	1:4	30	40:1	50	50	40, 50, 60, 70, 80

**Table 3:** Factors and levels in the Box–Behnken experimental design.

Levels	Factors			
	A: Liquid-to-Material Ratio (mL/g)	B: Time (min)	C: Temperature (°C)	D: Power (%)
−1	30:1	40	40	50
0	40:1	50	50	60
1	50:1	60	60	70

**Table 4:** The central composite design experimental schemes and results.

Number	A Liquid-to-Material Ratio (g/mL)	B Time (min)	C Temperature (°C)	D Power (%)	Total Phenolic Content (mg GAE/g DW)	Total Flavonoid Content (mg RE/g DW)	Score
1	−1	−1	0	0	12.00 ± 0.36	36.94 ± 0.25	25.38 ± 0.04
2	1	−1	0	0	15.92 ± 0.60	37.00 ± 0.35	27.23 ± 0.14
3	−1	1	0	0	15.19 ± 0.77	35.24 ± 0.11	25.95 ± 0.41
4	1	1	0	0	12.57 ± 0.19	28.05 ± 1.22	20.88 ± 0.87
5	0	0	−1	−1	11.66 ± 0.69	21.78 ± 0.21	17.09 ± 0.21
6	0	0	1	−1	14.28 ± 0.41	30.86 ± 0.55	23.18 ± 0.32
7	0	0	−1	1	14.49 ± 0.25	30.87 ± 0.16	23.28 ± 0.03
8	0	0	1	1	15.70 ± 0.30	35.90 ± 0.70	26.54 ± 0.39
9	−1	0	0	−1	12.21 ± 0.19	25.87 ± 0.20	19.54 ± 0.12
10	1	0	0	−1	10.21 ± 0.04	19.94 ± 0.36	15.43 ± 0.20
11	−1	0	0	1	12.40 ± 0.16	27.39 ± 0.07	20.44 ± 0.07
12	1	0	0	1	12.83 ± 0.47	29.66 ± 0.69	21.86 ± 0.38
13	0	−1	−1	0	16.63 ± 0.24	39.01 ± 0.62	28.63 ± 0.39
14	0	1	−1	0	13.87 ± 0.80	30.76 ± 0.24	22.93 ± 0.40
15	0	−1	1	0	17.07 ± 0.18	39.30 ± 0.35	29.00 ± 0.12
16	0	1	1	0	17.59 ± 1.01	44.40 ± 0.58	31.98 ± 0.43
17	−1	0	−1	0	13.21 ± 0.59	30.08 ± 0.40	22.27 ± 0.24
18	1	0	−1	0	11.74 ± 0.25	23.48 ± 0.24	18.04 ± 0.18
19	−1	0	1	0	15.02 ± 0.27	32.98 ± 0.39	24.66 ± 0.12
20	1	0	1	0	16.06 ± 0.44	37.23 ± 0.39	27.42 ± 0.41
21	0	−1	0	−1	15.40 ± 0.28	35.24 ± 0.11	26.04 ± 0.19
22	0	1	0	−1	12.14 ± 0.28	24.14 ± 0.48	18.58 ± 0.31
23	0	−1	0	1	16.23 ± 0.48	37.38 ± 0.49	27.58 ± 0.46
24	0	1	0	1	14.66 ± 0.51	32.56 ± 0.50	24.27 ± 0.50
25	0	0	0	0	18.25 ± 0.27	47.02 ± 0.84	33.69 ± 0.32
26	0	0	0	0	17.78 ± 0.20	47.18 ± 0.58	33.55 ± 0.43
27	0	0	0	0	18.12 ± 0.03	45.67 ± 0.96	32.90 ± 0.53
28	0	0	0	0	17.30 ± 0.43	45.74 ± 0.20	32.56 ± 0.26
29	0	0	0	0	17.44 ± 0.41	47.08 ± 0.48	33.34 ± 0.44

**Table 5:** The results of the variance analysis for the response surface fitting regression equation.

Source	Sum of Squares	Degrees of Freedom	Mean Square	F-Value	*p*-Value	Significance
model	763.38	14	54.53	66.83	˂0.0001	**
A	4.54	1	4.54	5.56	0.0334	*
B	30.98	1	30.98	37.96	˂0.0001	**
C	77.73	1	77.73	95.26	˂0.0001	**
D	48.41	1	48.41	59.34	˂0.0001	**
AB	11.96	1	11.96	14.66	0.0018	**
AC	12.22	1	12.22	14.97	0.0017	**
AD	7.65	1	7.65	9.38	0.0084	**
BC	18.86	1	18.86	23.11	0.0003	**
BD	4.31	1	4.31	5.29	0.0374	*
CD	2.00	1	2.00	2.45	0.1400	
A^2^	286.23	1.	286.23	350.80	˂0.0001	**
B^2^	19.29	1	19.29	23.64	0.0003	**
C^2^	75.11	1	75.11	92.06	˂0.0001	**
D^2^	345.77	1	345.77	423.76	˂0.0001	**
residuals	11.42	14	0.82			
lack of fit	10.54	10	1.05	4.77	0.0727	
pure error	0.88	4	0.22			
total sum	774.81	28				

Note: Asterisks indicate statistical significance, where “**” represents *p* < 0.0001 and “*” represents *p* > 0.05.

## Data Availability

The data presented in this study are available on request from the corresponding author.
